# Adult Computed Tomography examinations in Uganda: Towards determining the National Diagnostic Reference Levels

**DOI:** 10.1186/s12880-022-00838-x

**Published:** 2022-06-11

**Authors:** Geoffrey Erem, Faith Ameda, Caroline Otike, William Olwit, Aloysius G. Mubuuke, Cyril Schandorf, Akisophel Kisolo, Michael G. Kawooya

**Affiliations:** 1 Department of Radiology, School of Medicine, Makerere University, Kampala, Uganda; 2 Clinical Epidemiology Unit, School of Medicine, Makerere University, Kampala, Uganda; 3 Department of Nuclear Safety and Security, School of Nuclear and Allied Sciences, University of Ghana, Accra, Ghana; 4 Department of Nuclear Physics, Makerere University, Kampala, Uganda; 5 Department of Radiology, Ernest Cook Ultrasound Research and Education Institute (ECUREI), Kampala, Uganda

**Keywords:** Computed Tomography, Diagnostic Reference Level, Volume-weighted Computed Tomography Dose Index, Dose Length Product, Uganda

## Abstract

**Introduction:**

Diagnostic Reference Levels (DRLs), typically set at the 75th percentile of the dose distribution from surveys conducted across a broad user base using a specified dose-measurement protocol, are recommended for radiological examinations. There is a need to develop and implement DRLs as a standardisation and optimisation tool for the radiological protection of patients at Computed Tomography (CT) facilities.

**Methods:**

This was a retrospective cross-sectional study conducted in seven (7) different CT scan facilities in which participants were recruited by systematic random sampling. The study variables were dose length product (DLP) and volume-weighted CTDI (CTDIvol) for the radiation doses for head, chest, abdomen and lumbar spine CT examinations. The DRLs for CTDIvol and DLP were obtained by calculating the 3rd quartiles of the radiation doses per study site by anatomical region. The national diagnostic reference levels were determined by computation of DRLs using the 75th centile of the median values.

**Results:**

A total of 574 patients were examined with an average age of 47.1 years. For CTDIvol estimates; there was a strong positive significant relationship between the CTDIvol and examination mAs (r_s_ = 0.9017, *p*-value < 0.001), and reference mAs (r_s_ = 0.0.7708, *p*-value < 0.001). For DLP estimates; there was a moderate positive significant relationships between DLP and total mAs (r_s_ = 0.6812, *p*-value < 0.001), reference mAs (r_s_ = 0.5493, *p*-value < 0.001). The DRLs were as follows; for head CT scan – the average median CTDIvol was 56.02 mGy and the DLP was 1260.3 mGy.cm; for Chest CT, the CTDI volume was 7.82 mGy and the DLP was 377.0 mGy.cm; for the abdomen CT, the CTDI volume 12.54 mGy and DLP 1418.3 mGy.cm and for the lumbar spine 19.48 mGy and the DLP was 843 mGy.cm, respectively.

**Conclusion:**

This study confirmed the need to optimize the CT scan parameters in order to lower the national DRLs. This can be achieved by extensive training of all the CT scan radiographers on optimizing the CT scan acquisition parameters. Continuous dose audits are also advised with new equipment or after every three years to ensure that values out of range are either justified or further investigated.

## Background

Computed Tomography (CT) scan uses multi-detector technology, an important radiological diagnostic tool because it can supply rapid multiplanar and sub-millimetre images of the whole body. It is also documented that CT is a known significant contributor to the individual and population radiation exposure dose [1].

Without the knowledge of diagnostic reference levels (DRLs) from CT examinations, the optimal dose for patients undergoing CT examinations cannot be realised [2].

There is an estimated risk of cancer which is attributed to the use of diagnostic X-rays due to the stochastic effects of the X-rays [3, 4]. In order to avoid the risk of over exposure of the patients to diagnostic X-rays, there is a need to optimize the CT scan exposure parameters by the CT technologist [5, 6].

In 1990, the International Commission on Radiological Protection (ICRP) recommended the use of DRLs for patients undergoing radiological examinations [7]. It was intended to be a simple test to identify situations where the patient radiation dose levels were unusually high [8]. The ICRP emphasizes that DRLs “are not for regulatory or commercial purposes, not a dose constraint, and not linked to limits” [9]. DRLs are typically set at the 75th percentile of the dose distribution from a survey conducted across a broad user base using a specified dose-measurement protocol.

Medical exposure to ionizing radiation constitutes a significant exposure to the population in comparison to all the other sources. There has been a tremendous increase in ionising radiation from medical procedures today than during the early 1980s, due to higher utilization and increased CT access [10].

Other scholars have opined that CT scan doses vary considerably within and across facilities; the primary factors influencing dose variation are multi-phase scanning and institutional protocol choices. It is however not clear whether these are the same factors in play in resource-limited settings like Uganda [11].

There were wide variations in the CT radiation doses across the various facilities in the study conducted in Nigeria. In contrast, the Dose Length Product (DLP) was considerably higher, the volume-weighted Computed Tomography Dose Index (CTDIvol) were comparable to the international levels [12].

In a study by Kiror et al. in Kenya, it was found that the CT scan examination radiation exposure was broadly distributed between the facilities. This study, in turn, recommended the need to develop and implement DRLs as a standardisation and optimisation tool for the radiological protection of patients at all the CT facilities in Kenya [13, 14]. In addition, Korir et al. demonstrated that patient doses for brain, chest and abdomen examinations were above the international DRLs by factors of one to four [13]. This study further demonstrated that multi-slice CT elevates patient radiation dose, justifying the need for local optimised scanning protocols and institutional DRLs for dose management without affecting diagnostic image quality [13].

Egypt is one of the few African countries to have developed the National DRL. Their key findings revealed a consistent problem of higher CT scan ranges of the DRLs for DLP despite the lower DRLs for volume-weighted CTDI [15].

Wide variations of the mean weighted CTDI (CTDIw) and DLP values for similar CT scan examinations amongst Tanzanian hospitals were mainly attributed to the variations in CT scanning protocols and scanner types. The mean CTDI(w) values per examination for almost all hospitals were below the proposed DRLs, while the mean DLP values per examination were almost all above the proposed DRLs for all except one hospital. These were mainly influenced by the large scan length used in these studies. To achieve the required dose level for the establishment of the national DRLs, it was concluded that further investigation of optimization of scanning protocols was needed [2].

The establishment of the national DRL is critical in optimising unnecessary medical radiation exposure to patients; this was why the ICRP recommended the use of DRLs for patients. Though DRLs have been established and evaluated in many settings, such evaluations have periodically occurred in the developed world. There is a dearth of published literature on national DRLs from resource-constrained settings whose CT scan equipment varies considerably in technology from those in the developed world. This study, therefore, sought to explore and survey the DRLs in Uganda and set out reference levels.

## Materials and methods

### Study design

This was a retrospective cross-sectional study.

### Participating institutions

We drew a list of all the functional CT scan units at the time of the study, then purposively selected government and private facilities. We ensured that there was a representation of the urban/cities and upcountry towns CT facilities. We recruited patients from seven [7] accounting for 35% of the CT scan facilities in Uganda, the facilities drawn from the public and private facilities across Uganda. There was a total of 25 CT scanners in Uganda at the time of the study for a population of 48 million and 72% of these CT scans were in the private centres. We ensured that we also drew participating facilities from the urban/cities vs upcountry areas.

### Sampling and sample size estimation

The study participants were recruited by systematic random sampling whereby every third adult patients were recruited for common studies like head, chest and abdomen then consecutive sampling for lumbar spine CT scan examinations.

IAEA recommends a minimum of 10 patients and a maximum of 20 adult patients per radiation centre in describing CT dose characteristics. Patients for a routine head, chest, abdomen and lumbar CT examinations were recruited. A total of 574 adult patients were recruited during one year from seven hospitals with CT scan machines in Uganda.

### Data collection

Data was collected from the CT scan console using a piloted data collection tool. Piloted data was manually extracted from the CT scan console and entered into the data collection tool. There are no established radiation dose management systems and software in Uganda.

The quantitative data were entered into the Epi-Info database for analysis. None of the facilities had size-specific dose estimate (SSDE) capabilities.

### Study variables

The study variables were kVp, mAs, reference mAs, examination mAs, total mAs, slice thickness, scan length, scan time, DLP, CTDI_vol_ Effective dose for head, chest, abdomen and lumbar spine exams per study site. The Effective dose was computed as a product of the Dose Length Product (DLP) and different conversion factors denoted the k-factor for the different tissues and anatomical regions.

$$E=k\times DLP$$

where k is the DLP to effective dose conversion factor (mSv /mGy.cm). The conversion factors used were 0.0021 and 0.014 respectively [16]

Image quality assessment was also done using a 5-point scale International Atomic Energy Agency (IAEA) tool.

Each image was graded for overall quality by the radiologist on a 5-point scale as follows:

1 = Unacceptable (Extremely poor).

2 = Suboptimal (Below average).

3 = Acceptable (Average).

4 = High quality (Above average).

5 = Too little noise, too high quality (Excellent).

### Analysis plan

Data were exported to STATA version 15 for analysis. Baseline characteristics were summarised using means for numerical variables that were normally distributed and medians for numerical variables that were not normally distributed, and frequency and proportions for categorical variables and presented in the form of tables.

CT scan variables for different facilities were compared for each of the examinations by comparing their means, medians and proportions depending on the variable types these were also presented in the form of tables.

To determine the DRLs, three variables were considered namely; volume-weighted CTDI, DLP and effective dose. The reference levels were obtained by calculating the median (2nd quartile) and 3rd quartiles of the radiation doses per study site by anatomical region to determine the LDRLs This was done for facilities with a minimum number of 20 patients. The NDRL was determined by computing the 75th percentiles of the median values of the LDRLs and presented in the form of tables.

### Quality control

This was ensured using the following measures: The respective unit radiographers conducted daily, weekly and monthly dose calibrations. The regulatory agency, namely, the AEC, requires these tests to be conducted and documented for every CT scan facility. The regulator however performs yearly review assessment (enforcement) control tests on the machines. The data collection tool was also pre-tested and adjustments were made accordingly; data were collected and edited before entry into the software. Data sets were backed up regularly and stored, at the end of the study; the original data, data directory, final database and study analysis have been archived.

### Ethical considerations

Ethical approval to conduct the study was granted by the School of Medicine Research and Ethics Committee of Makerere University (Protocol No REC REF 2015-150). The Research and Ethics Committee also waived informed consent because there was no direct interaction with the patients during data collection. The study was also carried out in accordance with relevant guidelines and regulations according to the Helsinki declaration. There was utmost confidentiality with regard to the medical information that was collected; password-protected personal computer restricted access to data.

## Results

### CT scan characteristics

Most (6/7) of the CT scans were Siemens machines and the CT types were greatly varied. The majority of the CT scans were 16 slices scanners (4/7) with one 128-slice scanner. The earliest CT scan was installed in 2007 and the most recent was installed in 2018. The majority were in private facilities, with only 3/7 in the public facilities. There were also more facilities located in the urban/ cities 4/7 with the rest located in the upcountry centres. All the CT scan machines are installed and situated in the urban centers. (Table 1).

**Table 1:** CT Scan unit characteristics

CT scanner make	Type	Scan mode	Slice thickness	Year of installation	Public/private	Location
Siemens	Somatom Perspective	Helical	128	2014	Private	Urban
Siemens	Somatom Go	Axial	16	2018	Private	Urban
Siemens	Somatom Emotions	Axial	6	2017	Private	Urban
Siemens	Somalis	Axial	2	2007	Private	Upcountry
Siemens	Somatom Scope	Helical	16	2018	Public	Urban
Siemens	Somatom Emotions	Axial	16	2012	Public	Upcountry
Brightspeed	GE	Axial	16	2012	Private	Upcountry

### Patient demographic characteristics

A total of 574 patients were enrolled in this study with an average age of 47.1 years. The patients undergoing chest CT scan examination had the highest average age of 51.4 years, whereas participants for head examination had the least average age of 34 years. The majority of the participants across the different examinations were male, with an overall percentage of 55.6% (Table 2).

**Table 2:** The descriptive statistics of 574 patients in seven selected health facilities in Uganda

Characteristic	Measure				
	Head (n = 163)	Chest (n = 155)	Abdomen (n = 156)	Lumber spine (n = 100)	Total, n = 574
Age	34 (27, 58)	51.4 (18.1)	48 (17.2)	46.2 (18.7)	47.1 (18.8)
*Sex n (%)*					
Female	70 (27.5)	63 (24.7)	77 (30.2)	45 (17.6)	255 (44.4)
Male	93 (29.2)	92 (28.8)	79 (24.8)	55 (17.2)	319 (55.6)

### The description of CT scan variables across the health facilities for head, chest, abdominal and lumbar spine examinations.

#### Head CT scan examinations

A total of 163 patients were examined from the selected health facilities across Uganda. The majority (86.5%, n = 141) of the facilities used 130 kVp as their exposure, the median total mAs was 3519. The average examination mAs and reference values were 218.7 and 207.9 respectively. Facility A had the lower mean for total mAs of 851(801, 1218) and facility C had the highest medial total mAs of 4626 (4452, 4727).

The slice thicknesses ranged from 2.5 and 8 mm, with facility D having the highest thickness of 8 mm and facility G having the least of 1.25 mm. The average scan lengths and scan times were 188.4 mm and 45.6 s respectively and most (55.1%) of images had a quality of 4 followed by 5. (Table 3).

**Table 3:** CT scan characteristics for Head CT among 163 patients in 7 selected facilities

Facility	A (n = 25)	B (n = 23)	C (n = 23)	D (n = 21)	E (n = 22)	F (n = 24)	G (n = 22)	Total (N = 163)
*kVp n (%)*								
120							22 (100.0)	22 (13.5)
130	26 (100.0)	24 (100)	23 (100.0	21 (100.0)	22 (100.0)	25 (100.0)	–	141 (86.5)
Total mAs	851 (801, 1218)**	3628.5 (745.6)	4626 (4452, 4727)**	5506 (620.6)	3600.5 (3396, 4440)**	2626.6 (444.4)	–	3519 (2446, 4626)**
Exam mAs	151 (140, 166)**	185.4 (8.8)	221 (6.3)	232.3 (19.0)	211.9 (28.6)	240 (0)	297 (0)	218.7 (44.6)
Reference mAs	188 (9.6)	165.3 (21.5)	–	240 (0)	270 (0)	–	–	207.9 (45.5)
Scan length (mm)	146.0 (43.7)	262 (161, 301)**	223.6 (19.2)	182.5 (13.1)	253.6 (15.9)	173.7 (19.9)	104.6 (22.1)	188.4 (61.0)
Scan time (s)	16.4 (4.8)	37.9 (2.7)	66.9 (5.8)	87.2 (6.3)	65.5 (10.3)	29.3 (3.5)	22.1 (6.5)	45.6 (25.9)
*Slice thickness (mm) n (%)*								
1.25							1 (4.6)	1 (0.6)
2.5							21 (95.4)	21 (12.9)
4	9 (34.6)		23 (100.0)					32 (19.6)
4.8					22 (100.0)			22 (13.5)
5	17 (65.4)	24 (100)						41 (25.2)
6						25 (100.0)		25 (15.3)
8				21 (100.0)				21 (12.9)
*Image quality, n (%)*								
3	–	–	5 (21.7)	1 (5.0)	5 (22.7)	1 (4.0)		12 (7.7)
4	1 (5.0)	20 (83.3)	18 (78.3)	8 (40.0)	15 (68.2)	2 (8.0)	22 (100.0)	86 (55.1)
5	19 (95.0)	4 (16.7)		11 (55.0)	2 (9.1)	22 (88.0)		58 (37.2)

**median (25th, 75th percentile)

#### Chest CT scan examinations

A total of 155 patients were examined. The majority (89.7%, n = 139) of the facilities used a kVp of 130; the median total mAs was 2220, the average examination mAs and reference mAs were 55.8 and 68.4, respectively. Facility A and D had the lowest and highest means for total mAs 1283.1(289.6) and 4958 (1648.6), respectively, and F and C had the lowest and height medians of 1271(940, 1812) and 4400 (3658, 9224).

Most (56.8%) of the patients were imaged with a slice thickness of 5 mm, and the average scan lengths and scan times were 318.8 mm and 19.0 s, respectively. Nearly 50% of the images had a quality of 5 (Table 4).

**Table 4:** CT scan characteristics for chest CT among 155 patients in 7 selected facilities

Facility	A (n = 20)	B (n = 30)	C (n = 27)	D (n = 21)	E (n = 20)	F (n = 21)	G (n = 16)	Total (N = 155)
*kVp n (%)*								
120							16 (100.0)	16 (10.3)
130	20 (100.0)	30 (100.0)	27 (100.0)	21 (100.0)	20 (100.0)	21 (100.0)		139 (89.7)
Total mAs	1283.1 (289.6)	1781 (1325, 2884)**	4400 (3658, 9224)**	4958 (1648.6)	2772.5 (2215.5, 3728**)	1271 (940, 1812)**	–	2220 (1197, 3895)**
Exam mAs	59.0 (15.0)	54 (0)	56 (36, 88)	44.5 (16.3)	46.1 (10.9)	55 (45, 77)**	198.5 (151.5, 295)**	55.8 (23.8)
Reference mAs	78.5 (6.7)	52.8 (0)	78.9 (13.1)	61.9 (8.7)	70 (0)	72.9 (9.0)	–	68.4 (14.6)
Scan length (mm)	318.6 (56.1)	397.8 (108.3)	302.8 (48.4)	281.0 (36.8)	513 (0)	287.2 (48.1)	29.4 (19.8, 55.8)**	318.8 (134.2)
Scan time (s)	6.0 (0.9)	15.3 (0)	23.8 (5.5)	27 (24, 31)	21.7 (6.7)	12.8 (1.3)	6.6 (0.8)	19.0 (17.1)
*Slice thickness (mm) n (%)*								
1		30 (100.0)						30 (19.4)
2.5							16 (100.0)	16 (10.3)
3						21 (100.0)		21 (13.5)
5	20 (100.0)		27 (100.0)		20 (100)			88 (56.8)
*Image quality n (%)*								
2			2 (7.4)	1 (4.8)			1 (6.3)	3 (1.9)
3		7 (23.3)	5 (18.5)	1 (4.8)	8 (40.0)	2 (9.5)	10 (62.5)	24 (15.5)
4	1 (5.0)	12 (40.0)	14 (51.9)	11 (52.3)	5 (25.0)	5 (23.8)	5 (31.2)	58 (37.4)
5	10 (95.0)	11 (36.7)	6 (22.2)	38.1)	7 (35.0)	14 (66.7)	3.8 (1.6)	70 (45.2)

**median (25th, 75th percentile)

#### Abdominal CT scan examinations

A total of 156 patients were examined for abdominal CT scans in this study. The majority (83.3%, n = 130) of the facilities exposed their patients to a kVp of 130; the median total mAs, examination mAs and reference values were 6115.5, 98 and 116, respectively. Most (53.9%) of the patients had slice thickness of 3.75 mm, and the median scan lengths and scan times were 412.5 mm and 16.2 s, respectively. Most (60.6%) of the patients imaged had a quality of 5 characterized as excellent (Table 5).

**Table 5:** CT scan characteristics for abdomen CT among 156 patients in 7 selected facilities

Facility	A (n = 35)	B (n = 26)	C (n = 9)	D (n = 26)	E (n = 25)	F (n = 15)	G (n = 20)	Total (N = 156)
*kVp n (%)*								
110	6 (17.1)							6 (3.8)
120							20 (100.0)	20 (12.8)
130	29 (82.9)	26 (100)	9 (100.0)	26 (100.0)	25 (100.0)	15 (100.0)		130 (83.3)
Total mAs	3281 (2507, 4285)**	5685 (4271, 9659)**	12,173.6 (7493.7)	7721.5 (6208, 10,186)**	10,774 (9327, 13,647)**	3205 (2117, 4213)**	-	6115.5 (3619, 9869)**
Exam mAs	104 (82, 132)**	94.3 (12.0)	92.9 (28.8)	45 (14.9)	88 (73, 112)	83 (60, 111)**	315 (258, 315)**	98 (67, 118)**
Reference mAs	148 (4.1)	65.7 (21.3)	122 (4.4)	87.1 (10.5)	114.2 (8.2)	121.3 (3.5)	-	116 (80.5, 140)**
Scan length (mm)	421.5 (374.5, 444)**	643.5 (612, 705)**	371.5 (252.5, 400)**	360.2 (100.4)	759 (51.5)	347.2 (78.9)	60.9 (22.9, 115.1)**	412.5 (320, 633)**
Scan time (s)	12.3 (11.1, 13.8)**	41.2 (3.8)	28.7 (16.5)	34.1 (10.3)	22.7 (9.4)	16.9 (9.3)	11.9 (1.5)	16.2 (12.2, 34)**
Slice thickness (mm) n (%)								26 (16.7)
1		26 (100.0)						26 (16.7)
3				26 (100.0)				20 (12.8)
3.75							20 (100.0)	84 (53.9)
5	35 (100.0)		9 (100.0)		25 (100.0)	15 (100.0)		26 (16.7)
*Image quality n (%)*								
3		2 (7.7)	2 (22.2)	10 (38.5)		1 (7.1)		15 (9,7)
4		22 (84.6)	7 (77.8)	10 (38.5)	2 (8.0)	3 (21.4)	2 (10.0)	46 (29.7)
5	35 (100.0)	2 (7.7)		6 (23.0)	23 (92.0)	10 (71.4)	18 (90.0)	94 (60.6)

**median (25th, 75th percentile)

#### Lumbar spine examinations

A total of 100 patients were selected from 6 selected health facilities in Uganda, one facility did not have enough patient numbers to for recruitment in the study. The majority (98.0%, n = 98) of the facilities exposed their patients to a kVp of 130, the median total mAs was 5599.5, and the average examination mAs and reference values were 157.9 and 191.3, respectively. The majority (82.4%) of the patients had a slice thickness of 3 mm, and the median scan lengths and scan times were 348 mm and 54 s, respectively. Most (60.0%) of the images had a quality of 5 (Table 6).

**Table 6:** CT scan characteristics for lumbar spine CT among 100 patients in 7 selected facilities

Facility	A (n = 25)	B (n = 23)	C (n = 5)	D (n = 21)	F (n = 24)	G (n = 2)	Total (N = 100)
*kVp n (%)*							
120						2 (100.0)	2 (2.0)
130	25 (100.0)	23 (100.0)	5 (100.0)	21 (100.0)	24 (100.0)		98 (98.0)
Total mAs	2787 (1919, 3254)**	5147 (3802, 6140)**	5112 (4158, 5112)**	8389 (8037, 8863)**	6004 (5239, 8987)**		5599.5 (3277, 8083)**
Exam mAs	174 (134, 207)**	182 (0)	118 (20.9)	115 (21.9)	135 (107, 165.5)	277.5 (27.6)	157.9 (57.6)
Reference mAs	250 (250, 250)**	132 (115, 208)**	190 (0)	131 (28.5)	195.9 (42.1)		191.3 (52.8)
Scan length (mm)	381 (345, 444)**	333 (40.6)	387.7 (37.7)	302.3 (50.7)	397.2 (78.0)	90.3 (0)	348 (304.5, 407)**
Scan time (s)	20.7 (4.0)	25.8 (0)	71.7 (16.1)	56.4 (2.5)	66.3 (11.5)	51.5 (41.7)	54 (21.8, 59)**
*Slice thickness (mm) n (%)*							
1.25						2 (100.0)	2 (2.2)
1.5		14 (100.0)					14 (15.4)
3	25 (100.0)		5 (100.0)	21 (100.0)	24 (100.0)		75 (82.4)
*Image quality n (%)*							
3	1 (4.0)	1 (4.4)		7 (33.3)	1 (4.2)		10 (10.0)
4	3 (12.0)	10 (43.5)	1 (20 .0)	11 (52.4)	3 (12.5)	2 (100.0)	30 (30.0)
5	21 (84.0)	12 (52.1)	4 (80.0)	3 (14.3)	20 (83.3)		60 (60.0)

**median (25th, 75th percentile)

### Relationship between CT scan variables and diagnostic reference level estimates

#### For continuous CT scan variables

Overall, for CTDI vol estimates; there was a strong positive significant relationship between CTDI vol and examination mAs (r_s_ = 0.9017, *p*-value < 0.001) and reference mAs (r_s_ = 0.0.7708, *p*-value < 0.001). There was a weak negative significant relationship between scan length (r_s_ = − 0.3867, *p*-value < 0.001) and CTDI vol. The study also found a poor positive significant relationship between scan times (r_s_ = 0.1896, *p*-value < 0.0005) and volume CTDI**.**

For DLP estimates; there was a moderate positive significant relationship between DLP and total mAs (r_s_ = 0.6812, *p*-value < 0.001), reference mAs (r_s_ = 0.5493, *p*-value < 0.001), and a weak positive significant relationship between DLP, total mAs (r_s_ = 0.3693, *p*-value < 0.001), and scan length (r_s_ = 0.2342, *p*-value < 0.001) (Table 7).

**Table 7:** Spearman rank correlation coefficients for the relationship between the continuous CT scan variables and diagnostic reference level estimates

r_s_ (p-value)	Total mAs	Exam mAs	Reference mAs	Scan length (mm)	Scan times
*Overall*					
CTDI_vol mGy_	− 0.0843 (0.1249)	0.9017 (0.0000)*	0.7708 (0.0000)*	− 0.3867 (0.0000)*	0 1896 (0.0005)*
DLP mGy.cm	0.3693 (0.0000)*	0.6812 (0.0000)*	0.5493 (0.0000)*	0.2342 (0.0000)*	0 1074 (0.0502)
*Head CT*					
CTDI_vol mGy_	0.6543 (0.0000)*	0.8921 (0.0000)*	0.5546 (0.0000)*	0.4503 (0.0002)*	0.6328 (0.0000)*
DLP mGy.cm	0.6111 (0.0000)*	0.6786 (0.0000)*	0.1418 (0.2600)	0.3310 (0.0071)*	0.4767 (0.0001)*
*Chest CT*					
CTDI_vol mGy_	0.2597 (0.0084)*	0.7372 (0.0000)*	0.2245 (0.0233)*	− 0.2918 (0.0029)*	− 0.1089 (0.2759)
DLP mGy.cm	0.3484 (0.0003)*	0.4869 (0.0000)*	0.3630 (0.0002)*	0.3129 (0.0014)*	− 0.0002 (0.9986)
*Abdomen CT*					
CTDI_vol mGy_	− 0.0215 (0.8202)	0.8095 (0.0000)*	0.6664 (0.0000)*	0.1005 (0.2874)	− 0.3289 (0.0004)*
DLP mGy.cm	0.3480 (0.0001)*	0.7251 (0.0000)*	0.4453 (0.0000)	0.3973 (0.0000)*	− 0.1953 (0.0373)
*Lumbar spine CT*					
CTDI_vol mGy_	0.0104 (0.9416)	0.9388 (0.0000)*	0.5370 (0.0000)*	0.2350 (0.0935)	− 0.2543 (0.0688)
DLP mGy.cm	0.1148 (0.4179)	0.7602 (0.0000)*	0.4428 (0.0010)*	0.6172 (0.0000)*	− 0.0140 (0.9217)

*Significant relationship

#### Head CT scan

For volume CTDI estimates; there was a strong positive significant relationship for examination mAs (r_s_ = 0.8921, *p*-value < 0.001), a moderate positive significant relationship for total mAs (r_s_ = 0.6543, *p*-value < 0.001), reference mAs (r_s_ = 0.0.5546, *p*-value < 0.001) and scan times (r_s_ = 0.6328, *p*-value < 0.001). The scan length (r_s_ = 0.4503, *p*-value = 0.0002) had a weak positive relationship.

For DLP estimates, there was a moderate positive significant relationship for the total mAs (r_s_ = 0.6111, *p*-value < 0.001) and examination mAs (r_s_ = 0.6786, *p*-value < 0.001). This study also found a weak positive significant relationship for scan length (r_s_ = 0.3310, *p*-value = 0.0071) and scan times (r_s_ = 0.4767, *p*-value = 0.0001). (Table 7).

#### Chest CT scan

For CTDI vol estimates; there was a moderate positive significant relationship with the examination mAs (r_s_ = 0.7372, *p*-value < 0.001), and a weak positive significant relationship with the total mAs (r_s_ = 0.2597, *p*-value = 0.0084), reference mAs (r_s_ = 0.2245, *p*-value = 0.023) and a weak moderate significant relationship with the scan length (r_s_ = − 0.2918, *p*-value = 0.0029).

For DLP; there was a weak positive significant relationship with the total mAs (r_s_ = 0.3484, *p*-value = 0.0003), examination mAs (r_s_ = 0.4869, *p*-value < 0.001), reference mAs (r_s_ = 0.3630, *p*-value = 0.0002) and scan length (r_s_ = 0.3129, *p*-value = 0.0014) respectively (Table 7).

#### Abdominal CT scan

For CTDI vol; there was a strong positive significant relationship with the examination mAs (r_s_ = 0.8095, *p*-value < 0.001), a moderate positive significant relationship with the reference mAs (r_s_ = 0.6664, *p*-value < 0.001), and a weak negative significant relationship with scan times (r_s_ = − 0.3289, *p*-value < 0.0004).

For DLP; there was a moderate positive significant relationship with the examination mAs (r_s_ = 0.7251, *p*-value < 0.001), a weak positive significant relationship with total mAs (r_s_ = 0.3480, *p*-value = 0.0001) and scan length (r_s_ = 0.3973, *p*-value < 0.001) respectively (Table 7).

#### Lumbar spine CT

For CTDI vol; there was a strong positive significant relationship with the examination mAs (r_s_ = 0.9388, *p*-value < 0.001) and moderate positive significant relationship with reference mAs (r_s_ = 0.5370, *p*-value < 0.001).

For DLP; there was a moderate positive and significant relationships with examination mAs (r_s_ = 0.7602, *p*-value < 0.001) and scan length (r_s_ = 0.6172, *p*-value < 0.001) and a weak positive significant relationship with the reference mAs (r_s_ = 0.4428, *p*-value = 0.0010). (Table 7).

#### For categorical CT scan variables

Overall, there was a significant relationship between CTDI volume and DLP with kVp, slice thickness and image quality.

There was a significant relationship between CTDI volume and DLP with kVp, slice thickness and image quality for head CT scan. There was a significant relationship between DLP and slice thickness for chest CT scans.

For abdomen CT, there was a significant relationship between both CTDI volume and DLP with kVp, slice thickness and image quality. For lumbar spine CT, there was a significant relationship between CTDI volume and kVp, slice thickness and image quality, and DLP and image quality (Table 8).

**Table 8:** Kruskal–Wallis rank test for the relationship between categorical CT scan variables and Diagnostic Reference Level estimates

	kVp			Slice thickness (mm)		
	Chi-square	*p*-value	Degree of freedom	Chi-square	*p*-value	Degree of freedom
*Overall*						
CTDI_vol mGy_	10.578	0.0050**	2	246.021	0.0001**	10
DLP mGy.cm	21.127	0.0001**	2	106.396	0.0001**	10
*Head CT*						
CTDI_vol mGy_	56.744	0.0001**	1	115.026	0.0001**	6
DLP mGy.cm	46.365	0.0001**	1	74.473	0.0001**	6
*Chest CT*						
CTDI_vol mGy_	1.302	0.2539	1	2.752	0.4315	3
DLP mGy.cm	1.511	0.2190	1	18.838	0.0003**	3
*Abdomen CT*						
CTDI_vol mGy_	7.119	0.0284**	2	68.116	0.0001**	3
DLP mGy.cm	4.969	0.0834**	2	48.711	0.0001**	3
*Lumbar spine CT*						
CTDI_vol mGy_	4.176	0.0410**	1	9.991	0.0068**	2
DLP mGy.cm	3.688	0.0548	1	4.203	0.1223	2

**significant relationship

#### Diagnostic Reference Level (DRL) values

For head CT scan, the average median CTDI volume values across up to six selected facilities were 56.02 mGy, and the average median DLP value was 1260.3 mGy.cm. Facility A has the lowest CTDIvol and DLP, whereas facility F had the highest CTDIvol and DLP, respectively.

For chest CT scan, the average of the median CTDI volume values across the selected facilities was 7.82 mGy and the average of the median DLP value was 377.0 mGy.cm. Facility E had the lowest volume CTDI and facility A had the height, whereas, for DLP facility D had the lowest and facility C had the highest. (Table 9).

**Table 9:** CTDlvol and DLP values for Head and Chest CTs in 6 facilities

Facility	CTDIvol mGy				DLP mGy.cm				
	1st quartile	2nd quartile	3rd quartile	75th/25th percentile	1st quartile	2nd quartile	3rd quartile	75th/25th percentile	Effective dose
*Head (n* = *163)*									
A	32.88	35.31	38.53	1.17	636	673.5	871	1.37	1.6 (0.4)
B	39.25	42.04	48.245	1.23	800	1040	1232	1.54	2.1 (0.5)
C	51.92	51.92	51.92	1.00	1242	1291	1320	1.06	2.7 (0.2)
D	47.04	47.04	47.04	1.00	758	828	898	1.18	1.8 (0.2)
E	45.35	48.07	54.11	1.19	775	891.5	1042	1.34	1.9 (0.4)
F	57.38	57.38	57.38	1.00	1093.71	1168.31	1317.5	1.20	2.5 (0.3)
75th percentile (NDRL)	56.02				1260.33				2.1 (0.5)
*Chest (n* = *139)*									
A	6.25	8.01	10.76	1.72	239.95	298.09	469	1.95	4.1 (3.4, 6.6)**
B	5.36	6.23	8.38	1.56	182	246.5	399	2.19	3.5 (2.5, 5.6)**
C	4.49	7.23	11.46	2.55	289	356	721	2.49	5.0 (4.0, 10.1)**
D	5.76	6.98	8.92	1.55	172	218	278	1.62	3.1 (1.0)
E	3.97	5.05	5.69	1.43	293.5	384	408.5	1.39	5.2 (1.2)
F	5.11	6.95	9.51	1.86	158.79	213.9	309.17	1.95	3.8 (2.6)
75th percentile (NDRLs)	7.82				377				4.7 (2.8)

**median (25th, 75th percentile)

For abdominal CT scan has an overall median CTDI volume estimate of 12.54 mGy, the median DLP estimate was 1418.25 mGy.cm. Facility D had the lowest volume CVDI and A the highest, and facilities E and D had the highest and lowest DLP.

The lumbar spine was found to have an overall median CTDI volume estimate of 19.48 mGy and the median DLP estimate of 843.82 mGy.cm. Facility D and A had the lowest and highest CTDIvol and DLP, respectively (Table 10).

**Table 10:** CTDl and DLP values for Abdomen and Lumbar spine CTs in 4 facilities

Facility	CTDIvol mGy				DLP mGy.cm				
	1st quartile	2nd quartile	3rd quartile	75th/25th percentile	1st quartile	2nd quartile	3rd quartile	75th/25^th^ percentile	Effective dose
*Abdomen, n=132*									
A	8.37	12.56	17.96	2.15	945	1188	1601	1.69	19.7 (8.1)
B	6.41	7.56	10.18	1.59	590	789	1215	2.06	14.2 (6.2)
D	3.07	4.09	5.21	1.70	292	406	498	1.71	6.2 (1.2)
E	6.9	8.28	10.31	1.49	1129	1495	1685	1.49	25.8 (20.9)
75th percentile (NDRLs)	12.54				1418.25				15.6 (2.6)

Facility	CTDIvol mGy				DLP mGy.cm			
	1st quartile	2nd quartile	3rd quartile	75th/25th percentile	1st quartile	2nd quartile	3rd quartile	75th/25^th^ percentile
*Lumbar spine, n=98*								
Facility A	14.81	19.48	23.23	1.57	620	889.43	987	1.59
Facility B	14.87	19.48	26.39	1.77	515	707	849	1.65
Facility D	9.83	11.18	12.39	1.26	345	360	382	1.11
Facility F	13.55	17.03	20.91	1.54	583.73	703.66	925.11	1.58
75th percentile (NDRLs)	19.48				843.82			

#### Comparison of the DRLs with regional and international values

For Head CT scan, the CTDI volume in this study was 56 mGy compared to 52 mGy in Australia and 65 mGy France, and the DLP was 1260 mGy.cm compared to 810 mGy.cm in Turkey and 1612 mGy.cm in Kenya. For chest CT, the CTDI volume was 7.8 mGy compared to 10 mGy in Australia and 19 mGy in Kenya, and the DLP was 377 mGy.cm compared to 289 mGy.cm in Turkey and 895 mGy.cm in Kenya. The CTDI volume was 12.5 mGy compared to 12.3 mGy in the Ireland and 17 mGy in France for CT abdomen. The CTDI volume for lumbar spine examination in our study was 19.4 mGy compared to 20 mGy in Kenya and 45 mGy in France, and the DLP was 844 mGy.cm compared to 670 mGy.cm in Australia (Table 11).

**Table 11:** Comparison of the DRLS with regional and international values

EXAM	France 2015		Ireland 2012		Australia 2020		Turkey 2015		Kenya 2015		This study (Uganda 2022)	
	CTDIvol	DLP	CTDIvol	DLP	CTDIvol	DLP	CTDIvol	DLP	CTDIvol	DLP	CTDIvol	DLP
Head	65	1050	66.2	940	52	880	66.4	810	61	1612	56	1260
Chest	15	475	9.3	393	10	390	11.6	289	19	895	7.8	377
Abdomen	17	800	12.3	598	13	600	13.3	204	–	–	12.5	1418
Lumbar spine	45	700			26	670			20	769	19.4	844

## Discussion

The purpose of this study was to explore and survey the DRLs in Uganda and set out reference levels. A total of 574 patients were recruited from seven health facilities to determine the national diagnostic reference levels (NDRLs) for the head, chest, abdominal and lumbar spine CT examinations in Uganda. None of the facilities had size-specific dose estimate (SSDE) capabilities which is a method of estimating CT radiation dose that takes a patient's size into account.

The findings demonstrated that 75th percentiles of the’median values of the LDRLs were; volume-weighted CTDI values for head, chest, abdominal, and lumbar spine CT were 56.02, 7.82, 12.54 and 19.48 mGy, respectively. The average DLP values for head, chest, abdominal and, lumbar spine CT were 1260.33, 377.0, 1418.25 and 843.82 mGy.cm.

The average age of the patients was 47.1 years, with patients undergoing chest examination having the highest average age of 51.4 years, and those for head examination having the least average age of 34 years. The majority of the participants were male, with an overall percentage of 55.6%. Most of the patients usually presenting for CT are due to head trauma, whereby the young adult male is more commonly involved [13, 14]. The age of the patients for chest CT scan was higher probably because the study was conducted during the peak of the COVID pandemic where by the average age of the participants were the older people. However, this age profile is similar to the rest of the studies on COVID patients in Uganda [17, 18].

There were significant relationships between the CT scan variables and diagnostic reference level estimates namely the volume CTDI and DLP. DLP is a product of CTDI volume and scan length. DLP increased with an increase in scan length because DLP is a product of CTDI vol and scan length, hence there is a direct relationship between the two. When scan length increases, DLP will also increase. The relationship was weak probably due to tube loading technical aspects during the examination, but also other technical parameters include automatic exposure control systems (AEC) incorporated within CT scanners, varying tube current in our settings, pitch factor selections, and scan field of view.

For head CT, the volume CTDI increased with an increase in total mAs, examination mAs, reference mAs, scan length and the scan time. The DLP increased with an increase in total mAs, examination mAs, reference mAs and the scan times. The kVp, slice thickness and image quality influenced patients’ CTDI volume and DLP values.

For chest CT, the study found that the volume CTDI and the DLP increased with a increase in the total mAs, examination mAs, reference mAs and the scan length. The slice thickness also influenced the DLP.

For abdominal CT, the volume CTDI increased with an increase in examination mAs and reference mAs, and it increased as scan times reduced. The DLP increased with an increase in total mAs, reference mAs and the scan length. The study found that kVp, slice thickness and image quality influenced patients’ CTDI volume and DLP values.

For lumbar spine CT, the study found that the volume CTDI increased with an increase in examination mAs and reference mAs and the DLP increased with an increase in examination mAs, reference mAs and scan length. The study found that kVp, slice thickness and image quality influenced the CTDI volume and image quality influenced the DLP values of patients.

These findings are in tandem with the traditional scholarship and paradigm in CT scan radiation dosimetry. In optimising the DRL parameters namely the volume CTDI and DLP, emphasis needs to be put on the examination, total and reference mAs, scan length and scan time [5, 6]. This applies to the head, chest, abdominal and lumbar spine CT scan examinations.

The diagnostic reference levels (DRLs) of patients were established for different CT scan examinations and these were as follows. For head CT, the average volume CTDI and DLP values were 56.02 mGy and 1260.33 mGycm, respectively; the volume CTDI was lower in the majority of the studies but higher than in the Australian study of 2020; conversely, the DLP was higher than regional and international values except for a Kenyan study of 2015 [6, 12–17, 2–21]. For chest CT scan examination, the average volume CTDI and DLP estimates were 7.82 mGy and 377.0 mGycm respectively. The volume CTDI was lower than the regional and international studies; the DLP was lower than some of the African studies but comparable so a couple of European studies. For abdominal CT scan examination, the average volume CTDI and DLP estimates were 12.54 mGy and 1418.3 mGycm, respectively; the volume CTDI was lower than all the comparisons, however the DLP was way higher than other studies [6, 11–15, 19, 22–24]. The average volume CTDI and DLP estimates for lumbar spine CT were 19.48 mGy and 843.8 mGycm, respectively. The volume CTDI is comparable to the Kenyan study of 2015 but lower than the European comparisons. These DRL findings were higher than all the comparisons [6, 11–15, 19, 22]. This being the first nationwide study in Uganda, these variations are expected because of the differences in machines, lack of standardized protocols, wide variations in experience and training amongst the various CT radiographers and no National DRLs as a benchmark.

From this study, it will be incumbent upon the country to optimise the CT scan radiation doses. The dose reduction strategies to be adopted will include but are not limited to; scan time to be reduced to the order of several second, not several minutes, depending upon the imaging task, increasing the routine imaging thickness to 3–5 mm, adjusting kV taking into account patient size and effective use of AEC system if not yet activated.

## Conclusion and recommendation

The National Diagnostic References Levels have been determined for CT examinations for the head, chest, abdomen, and lumbar spine. There was a wide variation in the DRLs across facilities in Uganda due to differences in imaging protocols and equipment in use. The DRLs values for volume-weighted CTDI were comparable to the values obtained by other researchers. The DLPs values were markedly high across all the facilities and higher than the regional and the international values. Therefore, to optimise patient protection, optimised scanning protocols should be adopted by the facilities without sacrificing image quality.

## Data Availability

All the necessary data and materials have been included in this manuscript.
